# *HAM* Gene Family and Shoot Meristem Development

**DOI:** 10.3389/fpls.2021.800332

**Published:** 2021-12-20

**Authors:** Yuan Geng, Yun Zhou

**Affiliations:** ^1^Department of Botany and Plant Pathology, Purdue University, West Lafayette, IN, United States; ^2^Purdue Center for Plant Biology, Purdue University, West Lafayette, IN, United States

**Keywords:** shoot meristems, stem cells, land plants, HAM, GRAS proteins, microRNAs

## Abstract

Land plants develop highly diversified shoot architectures, all of which are derived from the pluripotent stem cells in shoot apical meristems (SAMs). As sustainable resources for continuous organ formation in the aboveground tissues, SAMs play an important role in determining plant yield and biomass production. In this review, we summarize recent advances in understanding one group of key regulators – the HAIRY MERISTEM (HAM) family GRAS domain proteins – in shoot meristems. We highlight the functions of HAM family members in dictating shoot stem cell initiation and proliferation, the signaling cascade that shapes HAM expression domains in shoot meristems, and the conservation and diversification of HAM family members in land plants. We also discuss future directions that potentially lead to a more comprehensive view of the *HAM* gene family and stem cell homeostasis in land plants.

## HAM Keeps Shoot Stem Cells Undifferentiated

**Figure 1:**
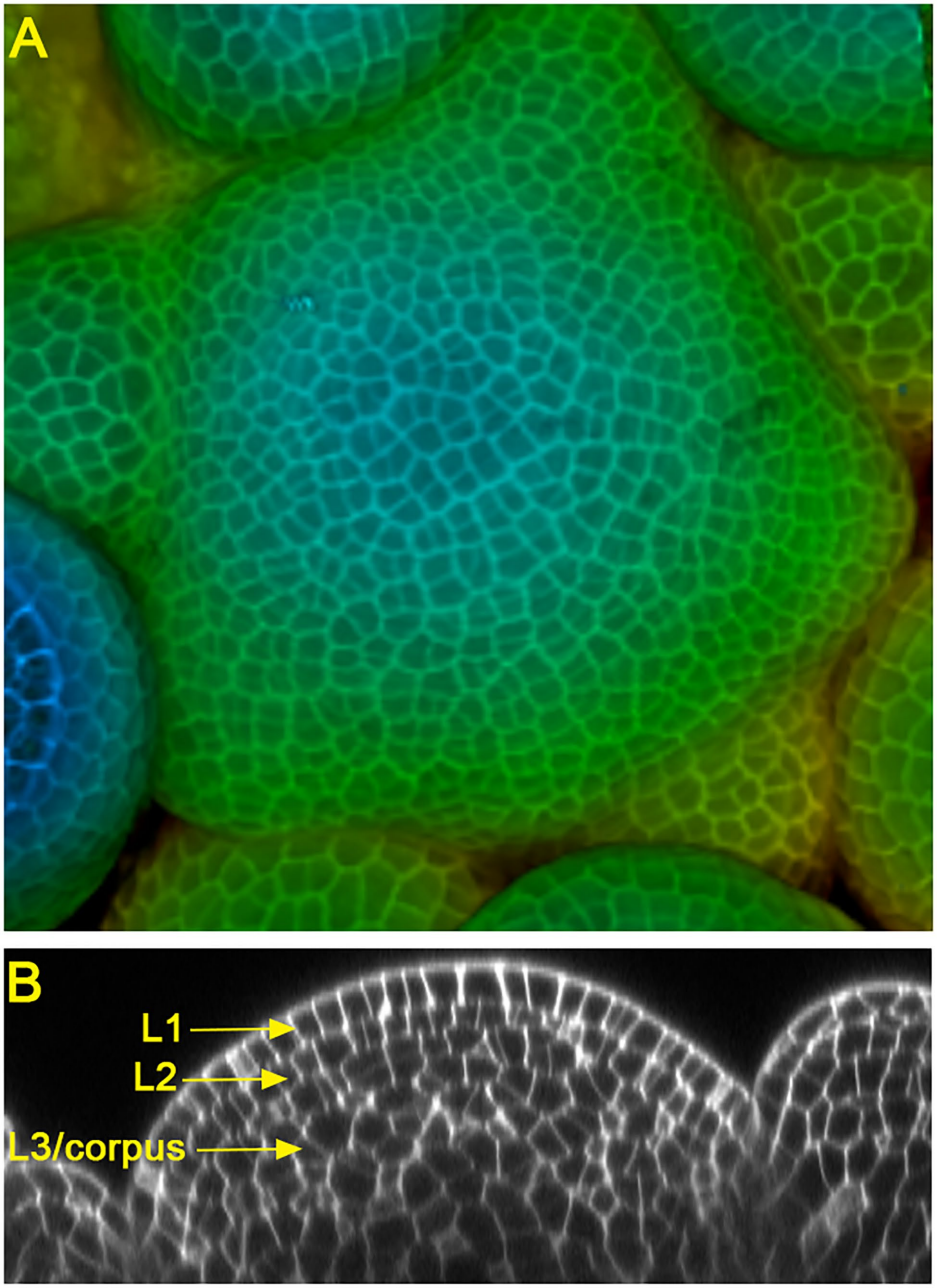
Confocal images of Arabidopsis inflorescence shoot apical meristems (SAMs). **(A)** The 3D projection view of an Arabidopsis SAM, with the depth color coding. Blue represents the top surface layer and red represents the deepest layer. **(B)** The orthogonal view of an Arabidopsis SAM, showing three clonally distinct cell layers: the epidermal layer (L1), sub-epidermal layer (L2), and corpus (L3).

Land plants develop diversified shoot architectures, which are determined and sustained by pluripotent stem cells in shoot apical meristems (SAMs). In seed plants, the multicellular SAMs are organized into distinct cell layers and zones (Foster, 1938; Satina et al., 1940; Meyerowitz, 1997). In the model species Arabidopsis and many other flowering plants, SAMs consist of three clonally distinct cell layers: the epidermal layer (L1), the sub-epidermal layer (L2), and the corpus (L3) (Figure 1). In addition, SAMs can be divided into different functional zones, including the central zone (CZ) where the self-renewing stem cells reside, the peripheral zone (PZ) where organ initiation takes place, and the rib meristem (RM) where the differentiated cells help specify the overlaying stem cells (Meyerowitz, 1997). Over more than 20 years of studies, multiple key regulatory pathways, such as the WUSCHEL-CLAVATA loop, KNOX/SHOOTMERISTEMLESS pathway, ERECTA family receptors, Class III HD-ZIP transcription factors, and the cytokinin and auxin signaling, have been identified and well characterized in Arabidopsis SAMs (Sablowski, 2007; Barton, 2010; Su et al., 2011; Shpak, 2013; Gaillochet and Lohmann, 2015; Somssich et al., 2016; Fletcher, 2018; Kieber and Schaller, 2018; Shi and Vernoux, 2021; Willoughby and Nimchuk, 2021). In this review, we focus on the function and regulation of one group of conserved stem cell regulators, the HAIRY MERISTEM (HAM) family GRAS (GAI, RGA, and SCR) domain proteins, in shoot meristems.

The *HAIRY MERISTEM* (*HAM*) gene was firstly identified and characterized in Petunia, and it was named after the phenotype of its loss-of-function mutant, the ectopic formation of differentiated hairs (trichomes) at the surface of shoot apices (Stuurman et al., 2002). The *HAM* loss-of-function in Petunia also shows early termination of SAMs, arrested axillary shoot development, and reduced number of carpels and stamens (Stuurman et al., 2002), suggesting the key role of HAM in maintaining shoot meristems undifferentiated in Petunia. In the model species Arabidopsis, four HAM homologs (HAM1-HAM4) are classified into two different groups, based on the phylogenetic analyses (Engstrom et al., 2011; Geng et al., 2021b). HAM1, HAM2, and HAM3, which are also named as LOST MERISTEM1 (LOM1), LOM2, and LOM3, respectively (Schulze et al., 2010), belong to the Type II group (Engstrom et al., 2011; Geng et al., 2021b). These Type II members (*HAM1-3*) are expressed in Arabidopsis shoot meristems, root meristems, and vascular tissues (Schulze et al., 2010; Engstrom et al., 2011; Zhou et al., 2015). HAM4, the only member of the Type I group in Arabidopsis (Engstrom et al., 2011; Geng et al., 2021b), is specifically expressed in the provascular and vascular tissues (Zhou et al., 2015), sharing redundant function with HAM1-3 during shoot and root development (Engstrom et al., 2011; Zhou et al., 2015).

The Type II HAM members (HAM1, HAM2, and HAM3) play both overlapping and distinct roles in control of Arabidopsis SAMs. The single loss-of-function mutant of each Type II member does not result in any obvious defects in Arabidopsis shoot meristem development (Schulze et al., 2010; Engstrom et al., 2011). By contrast, the *ham1ham2ham3* (*ham123*) triple loss-of-function mutant or the *ham1ham2* (*ham12*) double mutant showed delayed inflorescence initiation, early termination of shoot meristems, disorganized meristem structure and morphology, and reduced axillary shoot branches (Schulze et al., 2010; Wang et al., 2010; Engstrom et al., 2011; Han et al., 2020a), demonstrating essential and redundant roles of Type II members in meristem initiation and maintenance in Arabidopsis. A recent study further shows that HAM1 and HAM2, both of which are expressed in the L3 layer, are required for maintaining SAMs undifferentiated and driving *de novo* formation of new axillary stem cell niches (Han et al., 2020a). HAM3, the other member of the Type II group, plays a minor role in shoot stem cell maintenance but likely contributes to other aspects of shoot development (Han et al., 2020a).

## HAM Sustains the WUSCHEL-CLAVATA Regulatory Loop

**Figure 2:**
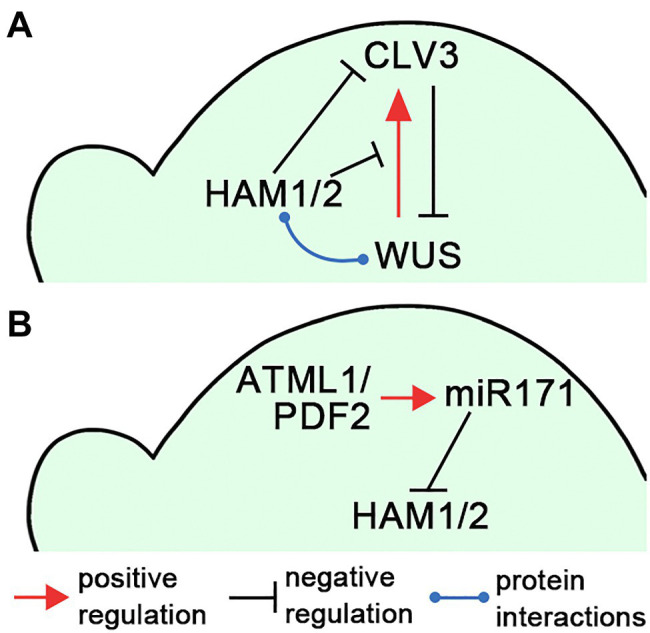
Illustrations of the HAM regulatory circuits in SAMs. **(A)** A diagram illustrates that HAM1/2 sustain the WUS-CLV3 feedback loop in Arabidopsis SAMs. **(B)** A diagram illustrates the L1(ATML1/PDF2)-miR171-HAM signaling cascade, which shapes HAM1/2 expression patterns in Arabidopsis SAMs. The positive and negative regulations and protein–protein interactions are indicated in **(A,B)**.

In Arabidopsis, the homeobox domain transcription factor WUSCHEL (WUS) and the secreted peptide CLAVATA3 (CLV3) form a negative feedback loop to keep a constant population of stem cells in SAMs (Schoof et al., 2000; Somssich et al., 2016; Fletcher, 2018; Figure 2A). The *WUS* transcripts are restricted into the organizing center (OC) in deep cell layers (Mayer et al., 1998) and WUS proteins move into stem cells in the central zone to activate *CLV3* expression (Schoof et al., 2000; Yadav et al., 2011; Daum et al., 2014). On the contrary, the CLV3 peptide, secreted from stem cells, activates the CLV receptor signaling pathways and confines *WUS* transcripts to the OC to avoid overproliferation of stem cells (Schoof et al., 2000). The ability of WUS to directly activate its own inhibitor *CLV3* brings a potential risk to shut down itself and the feedback loop; therefore, the precise spatial–temporal regulations of *WUS* and *CLV3* are required for stem cell maintenance.

Several studies demonstrated that Type II HAM members play essential roles in initiating and maintaining the WUS-CLV3 feedback loop, and further sustaining shoot stem cell homeostasis in Arabidopsis (Schulze et al., 2010; Zhou et al., 2015, 2018; Gruel et al., 2018; Han et al., 2020a; Geng et al., 2021b), which also has been summarized in the reviews (Biedermann and Laux, 2018; Han et al., 2020b). Through the screening of an Arabidopsis transcription factor library, Type II HAM proteins are identified as the WUS interacting partners (Zhou et al., 2015). Among them, both *HAM1* and *HAM2* are co-expressed with *WUS* in the L3 layer. HAM1/2 act as WUS transcriptional cofactors to regulate the downstream targets and drive proliferation of shoot stem cells (Zhou et al., 2015). In addition, the expression patterns of HAM1/2 and *CLV3* are largely complementary in Arabidopsis SAMs (Zhou et al., 2018). *CLV3* is highly expressed in the L1 and L2 layers of the central zone, where HAM1 and HAM2 are absent or barely detectable (Zhou et al., 2018; Han et al., 2020a). These results lead to a hypothesis that HAM1/2 together with WUS determine the *CLV3* expression pattern and confine the *CLV3* domain to the stem cells in the outer layers of SAMs (Zhou et al., 2018; Han et al., 2020b; Figure 2A). Specifically, WUS protein activates *CLV3* in the central zone where HAM1/2 proteins are absent, and HAM1/2 keep *CLV3* off in the rib meristem, preventing WUS-dependent activation of *CLV3* and/or repressing *CLV3* transcription (Zhou et al., 2018). This working model has been supported by (Zhou et al., 2018; Han et al., 2020a; Geng et al., 2021b) and aligns with (Brand et al., 2000, 2002; Schoof et al., 2000; Graf et al., 2010; Schulze et al., 2010) a number of experimental results. It is also shown plausible by several independent computational simulations (Gruel et al., 2018; Zhou et al., 2018; Liu et al., 2020). In addition, through confocal imaging of HAM translational reporters and genetic complementation analyses, recent work shows that both HAM1 and HAM2 proteins, which show highly comparable expression patterns in the L3 layer of SAMs, are necessary and sufficient for determining the *CLV3* pattern (Han et al., 2020a). HAM3, which is only expressed in the boundary between the meristem and primordia and at a few cells of the peripheral zone, is dispensable in shaping the *CLV3* domain (Han et al., 2020a). In contrast, when HAM3 is expressed in the rib meristem under the control of the *HAM2* promoter, it rescues the ectopic expression of *CLV3* in the *ham123* triple mutant (Han et al., 2020a), suggesting HAM3 protein maintains the function interchangeable with that of HAM1 and HAM2.

During the *de novo* formation of shoot stem cell niches, the expression patterns of HAM1/2 are dynamically regulated, which drive the switch of the *CLV3* expression domain from the basal to apical region of developing axillary meristems over time (Zhou et al., 2018). In contrast, the expression of *CLV3* is restricted to the basal part of developing axillary meristems in the *ham123* mutant, consistent with the mutant defects in axillary bud initiation (Schulze et al., 2010; Wang et al., 2010; Engstrom et al., 2011; Zhou et al., 2018).

## A Signaling Cascade Shapes HAM Patterns in Arabidopsis Shoot Meristems

In Arabidopsis, a small group of micro RNAs – the microRNA171 (miR171) – function as the negative regulator of Type II HAM members (Llave et al., 2002; Rhoades et al., 2002; Schulze et al., 2010; Wang et al., 2010; Engstrom et al., 2011; Han et al., 2020c). miR171 specifically recognizes and binds to Arabidopsis *HAM1, HAM2*, and *HAM3*, mediating the cleavage of their transcripts (Llave et al., 2002; Rhoades et al., 2002). Consistently, *MIR171* overexpression leads to ectopic expression of *CLV3* in the rib meristem and reduced shoot branching, which mimic the phenotype of the *ham123* mutant (Schulze et al., 2010; Wang et al., 2010; Zhou et al., 2018; Han et al., 2020a).

The epidermis-derived miR171 defines the apical-basal concentration gradient of HAM1/2 in Arabidopsis SAMs and axillary meristems (Takanashi et al., 2018; Han et al., 2020c). Four *MIR171* family genes (*MIR171A*, *MIR171B*, *MIR171C*, and *MIR170*) are identified in Arabidopsis, all producing miR171 precursors and contributing to the total level of mature miR171 (Llave et al., 2002; Rhoades et al., 2002). All these *MIR171/170* genes are directly activated by the homeodomain transcription factor ARABIDOPSIS THALIANA MERISTEM LAYER 1 (ATML1) and its close homolog PROTODERMAL FACTOR 2 (PDF2) in the L1 layer (Han et al., 2020c). Once synthesized in the epidermis, mature miR171 moves downwards within limited distance and it mediates the cleavage of the transcripts of *HAM1-3* in the apical region of SAMs (Han et al., 2020c). Based on these results, a L1(ATML1/PDF2)-miR171-HAM signaling cascade has been proposed, which initiates and then maintains the apical-basal concentration gradient of Type II HAM proteins in Arabidopsis shoot meristems (Han et al., 2020c; Figure 2B). The essential function of the L1-miR171-HAM signaling cascade is simulated by a computational model and further validated by *in vivo* experimentations including the time-lapse live imaging upon the transient activation of ATML1 in the SAMs (Han et al., 2020c).

## Evolution Of *HAM* Gene Family in Land Plants

The phylogenetic analysis suggested that the *HAM* gene family emerged during the divergence of land plant lineages (Geng et al., 2021b). In non-flowering plants including bryophytes, lycophytes, ferns, and gymnosperms, *HAM* members are maintained with a low copy number (Engstrom et al., 2011; Geng et al., 2021b). By contrast, the *HAM* gene family likely duplicated in a common ancestor of flowering plants, expanding to two diversified groups (Type I and Type II) as mentioned above, in flowering plants (Geng et al., 2021b). Type II HAM members are widely present in flowering plants, whereas Type I HAM members were independently lost in the species from different orders (including Poales and Asparagales) in monocots (Geng et al., 2021b).

HAM family members from several flowering plants share similar functions in maintaining indeterminacy of SAMs and promoting *de novo* formation of axillary meristems (Stuurman et al., 2002; Schulze et al., 2010; Wang et al., 2010; Engstrom et al., 2011; David-Schwartz et al., 2013; Zhou et al., 2015, 2018; Hendelman et al., 2016). For example, the *ham* loss-of-function mutant in pepper (*Capsicum annuum*) shows the shoot meristem defect (David-Schwartz et al., 2013) comparable to that characterized in the Petunia *ham* mutant and in the Arabidopsis *ham123* mutant (Stuurman et al., 2002; Schulze et al., 2010; Engstrom et al., 2011). Several HAM homologs, including AmHAM1 (the Type I) and AmHAM2 (the Type II) from *Amborella trichopoda* (the species as a sister group to all other flowering plants), one Type II HAM from a monocot (rice), and two Type II HAM members from eudicots (soybean and pepper), are able to replace the role of Arabidopsis Type II HAM members in Arabidopsis shoot meristems (Geng et al., 2021b), demonstrating the conserved function of HAM family members in flowering plants.

The results from cross-species complementation assays also indicate the conserved biochemical function between the non-flowering HAM proteins and the Type II HAM proteins from flowering plants, in regulating meristem development (Geng et al., 2021b). When different non-flowering *HAM* members (including *PpHAM* from the bryophyte *Physcomitrium* (*Physcomitrella*) *patens*, *SmHAM* from the lycophyte *Selaginella moellendorffii*, *CrHAM* from the fern *Ceratopteris richardii*, and *LkHAM* from the gymnosperm *Larix kaempferi*) are expressed under the control of the Arabidopsis *HAM2* promoter, they replace the function of Type II members (*HAM1*, *HAM2*, and *HAM3*) in regulating the *CLV3* expression domain, maintaining established SAMs, and promoting the initiation of new stem cell niches in Arabidopsis *ham123* mutants (Geng et al., 2021b). Consistently, the function of *PpGRAS12*/*PpHAM* was also characterized in the moss *Physcomitrium* (*Physcomitrella*) *patens* (Beheshti et al., 2021). Overexpression of *PpGRAS12* leads to formation of supernumerary apical meristems on each gametophore, suggesting a positive role of *PpGRAS12*/*PpHAM* in control of stem cell population at the gametophyte stage (Beheshti et al., 2021). Taken together, all the current results lead to a hypothesis that regulation of stem cell homeostasis is an ancestral and conserved trait of the *HAM* gene family, which deserves more functional studies of *HAM* homologs in land plants, especially in seed-free plants. Recent advances in the genomic and transcriptomic resources (Marchant et al., 2019; Geng et al., 2021a), established transformation system (Plackett et al., 2014) and quantitative confocal imaging platform (Wu et al., 2021) in seed-free vascular plants, such as in *Ceratopteris richardii*, will facilitate us to test this hypothesis and further understand meristem evolution in land plants.

## Conservation and Diversification of the *miR171-HAM* Regulation in Land Plants

The phylogenetic analysis and sequence alignment demonstrate that the 21-nt miR171 binding site (5′-GATATTGGCGCGGCTCAATCA-3′) is highly conserved within the coding sequences of the non-flowering *HAM* members and the majority of Type II *HAM* members in flowering plants (Engstrom et al., 2011; Geng et al., 2021b). The negative regulation of Type II *HAM* members by miR171 seems to be conserved in flowering plants as well. For example, transcripts of two *HAM* family genes (*SlHAM1* and *SlHAM2*) in tomato (*Solanum lycopersicum*) and four *HAM* homologs in rice (*Oryza sativa*) are also specifically targeted and cleaved by miR171 (Fan et al., 2015; Hendelman et al., 2016). Overexpression of *MIR171* genes in tomato and rice results in reduced expression of these *HAM* homologs and the disruption of meristem development (Fan et al., 2015; Hendelman et al., 2016).

Furthermore, when the non-flowering HAM members (such as *PpHAM*, *SmHAM*, *CrHAM*, and *LkHAM*) and several Type II HAM members from flowering plants (including *Amborella*, the monocot rice, and the dicot soybean and pepper) are expressed under the control of Arabidopsis *HAM2* promoter, these HAM reporters showed the concentration gradient from low to high along the apical-basal axis of Arabidopsis SAMs (Geng and Zhou, 2021; Geng et al., 2021b). These expression patterns are largely comparable to that of the miR171-sensitive *HAM2* translational reporter (Han et al., 2020a; Geng et al., 2021b); however, they are different from that of the miR171-insenstive *HAM2* transcriptional reporter, which shows high expression in all the cells from different layers in Arabidopsis SAMs (Han et al., 2020a). These findings suggest a conserved role of the miR171 binding sites in the non-flowering HAM members and in the majority of Type II HAM members from flowering plants.

Different from the Type II, Type I *HAM* genes show different extents of diversification in the miR171 binding site (Engstrom et al., 2011; Geng et al., 2021b). Based on the sequence alignment (Geng et al., 2021b), only a few Type I *HAM* members (such as *AmHAM1* from *Amborella trichopoda* and the *HAM* homologs from *Nelumbo nucifera* and *Vitis vinifera*) maintain the conserved miR171 binding site, and many others from a considerable number of flowering plants lost the conservation of the miR171 binding site. For example, *HAM4* (the Arabidopsis Type I *HAM*) contains six nucleotides different from the conserved miR171 binding sequence and is unlikely targeted by miR171 (Engstrom et al., 2011; Geng et al., 2021b).

## Future Perspectives

Over the last several years, significant progress has been made in understanding the functions of Type II HAM members in shoot meristems and their interaction with the WUS-CLV3 loop, the regulatory mechanism by which Type II HAM proteins are excluded from stem cells in Arabidopsis SAMs, and evolution of different groups of HAM members in land plants. In the future, several important questions are still remaining to be explored. For example, in Arabidopsis SAMs, in contrast to *WUS* and *CLV3* that are specifically expressed in a few cells, HAM1 and HAM2 proteins are expressed in a broader domain (Zhou et al., 2015, 2018; Han et al., 2020a). It will be interesting to explore whether the Type II HAM members also integrate additional and multiple regulatory pathways in control of shoot stem cells. In addition, the L1-miR171-HAM signaling cascade plays a crucial role during the initiation and maintenance of Arabidopsis shoot meristems (Han et al., 2020c). It will be worth determining whether this signaling cascade also functions in other meristematic tissues in Arabidopsis and whether this regulatory mechanism is conserved across flowering plants or even in non-flowering plants. Furthermore, the function of Type I HAM members is not completely understood yet. Determining whether and how this group of HAM members have been recruited into various developmental processes and undergone neofunctionalization in land plants will be an essential question in the future.

## Author Contributions

All authors listed have made a substantial, direct and intellectual contribution to the work, and approved it for publication.

## Funding

This work was supported by the Purdue start-up package and National Science Foundation IOS-1931114 (to YZ), and the Purdue Center for Plant Biology Graduate Student Fellowship and Ross-Lynn Graduate Research Scholarship (to YG).

## Conflict of Interest

The authors declare that the research was conducted in the absence of any commercial or financial relationships that could be construed as a potential conflict of interest.

## Publisher’s Note

All claims expressed in this article are solely those of the authors and do not necessarily represent those of their affiliated organizations, or those of the publisher, the editors and the reviewers. Any product that may be evaluated in this article, or claim that may be made by its manufacturer, is not guaranteed or endorsed by the publisher.
